# Supplementary material for: Hypothalamic neuronal circuits regulating hunger-induced taste modification
Source: Nat Commun. 2019 Oct 8;10:4560. doi: 10.1038/s41467-019-12478-x (PMC6783447; doi:10.1038/s41467-019-12478-x)
Supplement: Supplementary file 1 — Supplementary Information [file 41467_2019_12478_MOESM1_ESM.pdf]

Supplementary Information

Hypothalamic neuronal circuits regulating hunger-induced taste modification

Fu *et al*.

Supplementary Fig. 1

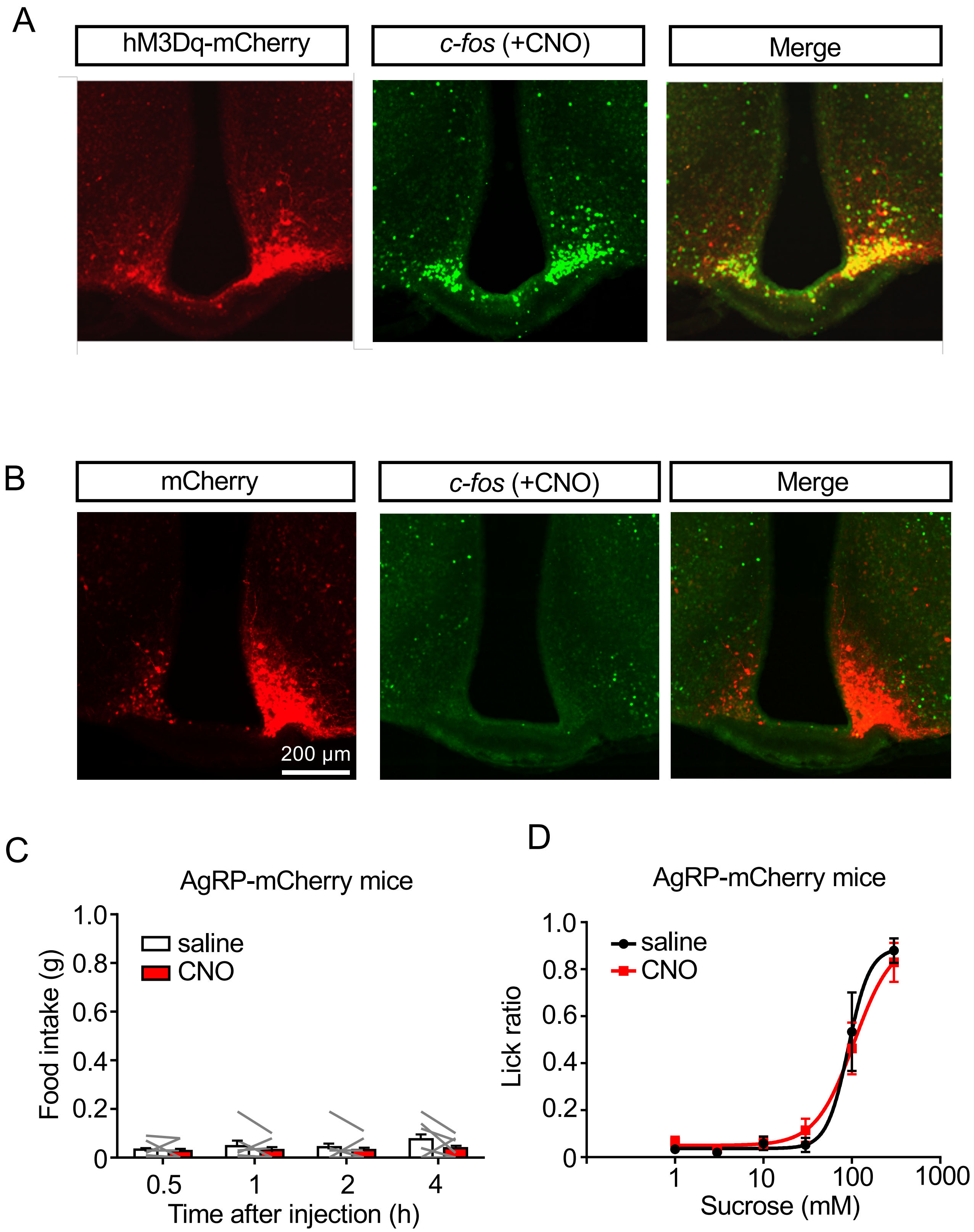

Supplementary Fig. 1 CNO induce *c-fos* expression in AgRP-hM3Dq-mCherry mice but has no effect on *c-fos* expression, food intake, and taste preference in AgRP-ires-Cre mice injected with the control AAV encoding Cre-dependent mCherry.

(A) *c-fos* expression in the ARC of AgRP-hM3Dq-mCherry mice 1 h after CNO injection (1.0 mg/kg i.p.). (B) AgRP-ires-Cre mice were injected with AAV-DIO-mCherry (AgRP-mCherry mice). Representative immunohistochemical image shows little c-Fos expression in the ARC of AgRP-mCherry mice 1 h after CNO injection (1.0 mg/kg i.p.). (C) Food intake in AgRP-mCherry mice. Acute feeding was not triggered by CNO treatment (1.0 mg/kg i.p.) in AgRP-mCherry mice. n=7, paired Student's *t*-test. (D) No difference was observed in preference for sucrose taste in AgRP-mCherry mice treated with CNO (1.0 mg/kg i.p.). n=7, F=0.003, P=0.9565, two-way ANOVA with Bonferroni *post hoc* test. All experiments were carried out with 10- to 16- week-old male mice. Data are given as means ± SEM.

Supplementary Fig. 2

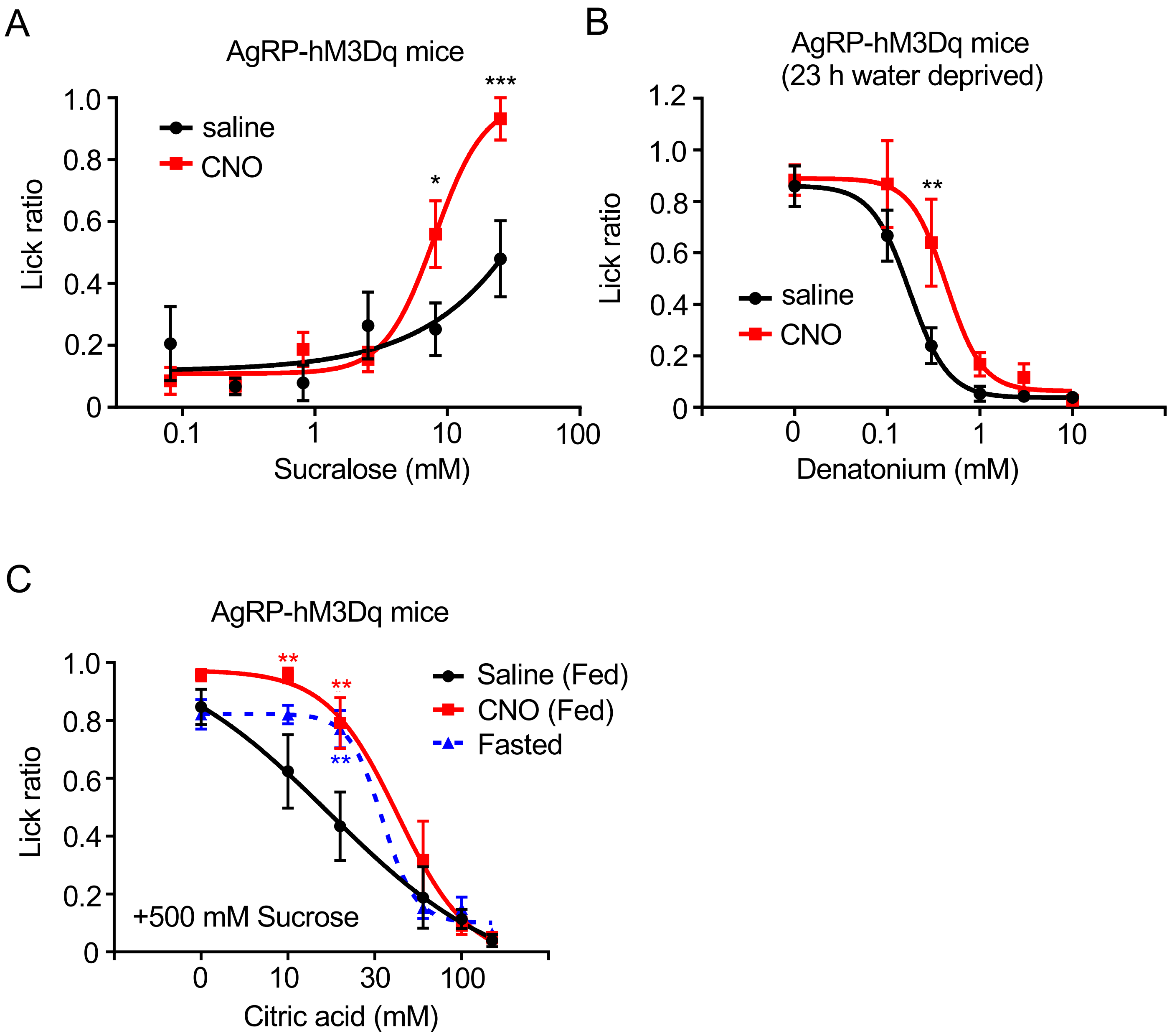

Supplementary Fig. 2 Chemogenetic activation of AgRP neurons also modifies taste preference towards sucralose and citric acid.

(A) Taste preference toward the non-calorie sweetener, sucralose, in AgRP-hM3Dq mice treated with saline or CNO (1.0 mg/kg i.p.). n=6, *F*=5.6, *P*=0.021, two-way ANOVA with Bonferroni *post hoc* test. (B) Taste preference toward denatonium in 23 h water deprived AgRP-hM3Dq mice treated with saline or CNO (1.0 mg/kg i.p.). n=6, *F*=7.98, *P*=0.0064, two-way ANOVA with Bonferroni *post hoc* test. (C) Taste preference toward citric acid in saline-treated fed AgRP-hM3Dq mice (black), CNO-treated (1.0 mg/kg i.p.) fed AgRP-hM3Dq mice (red), or overnight-fasted AgRP-hM3Dq mice (blue). n=8, *F*=7.45, *P*=0.0009, two-way ANOVA with Bonferroni *post hoc* test. All experiments were carried out with 10- to 16-week-old male mice. Data are given as means ± SEM. *P<0.05, **P<0.01, ***P<0.001 as compared to saline group.

Supplementalry Fig. 3

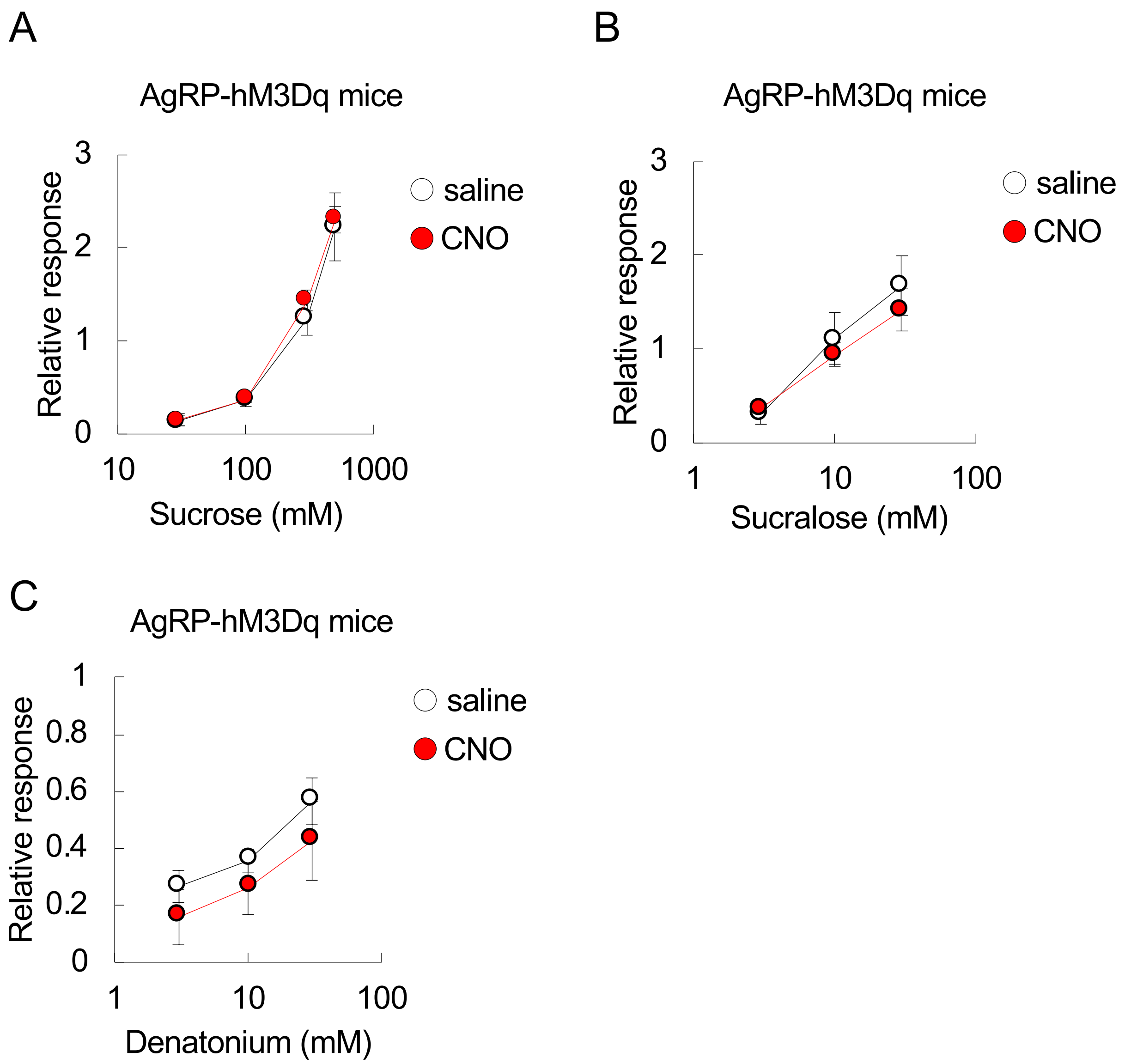

Supplementary Fig. 3 Chorda tympani nerve responses to gustatory stimuli are unaffected by chemogenetic activation of AgRP neurons in AgRP-hM3Dq mice.

Chorda tympani nerve response (relative to 100 mM NH_4Cl) for sucrose (A), sucralose (B), and denatonium (C) in AgRP-hM3Dq mice after either saline or CNO treatment. Mice were anesthetized using pentobarbital and nerve recording to taste solution was performed for 10 min after single injection of saline or CNO (1.0 mg/kg i.p.). Taste sensitivities for sweet and bitter tastants were not affected by the chemogenetic activation of AgRP neurons. All experiments were carried out with 10- to 20- week-old male mice (n=5–6 per group). Data are given as means ± SEM.

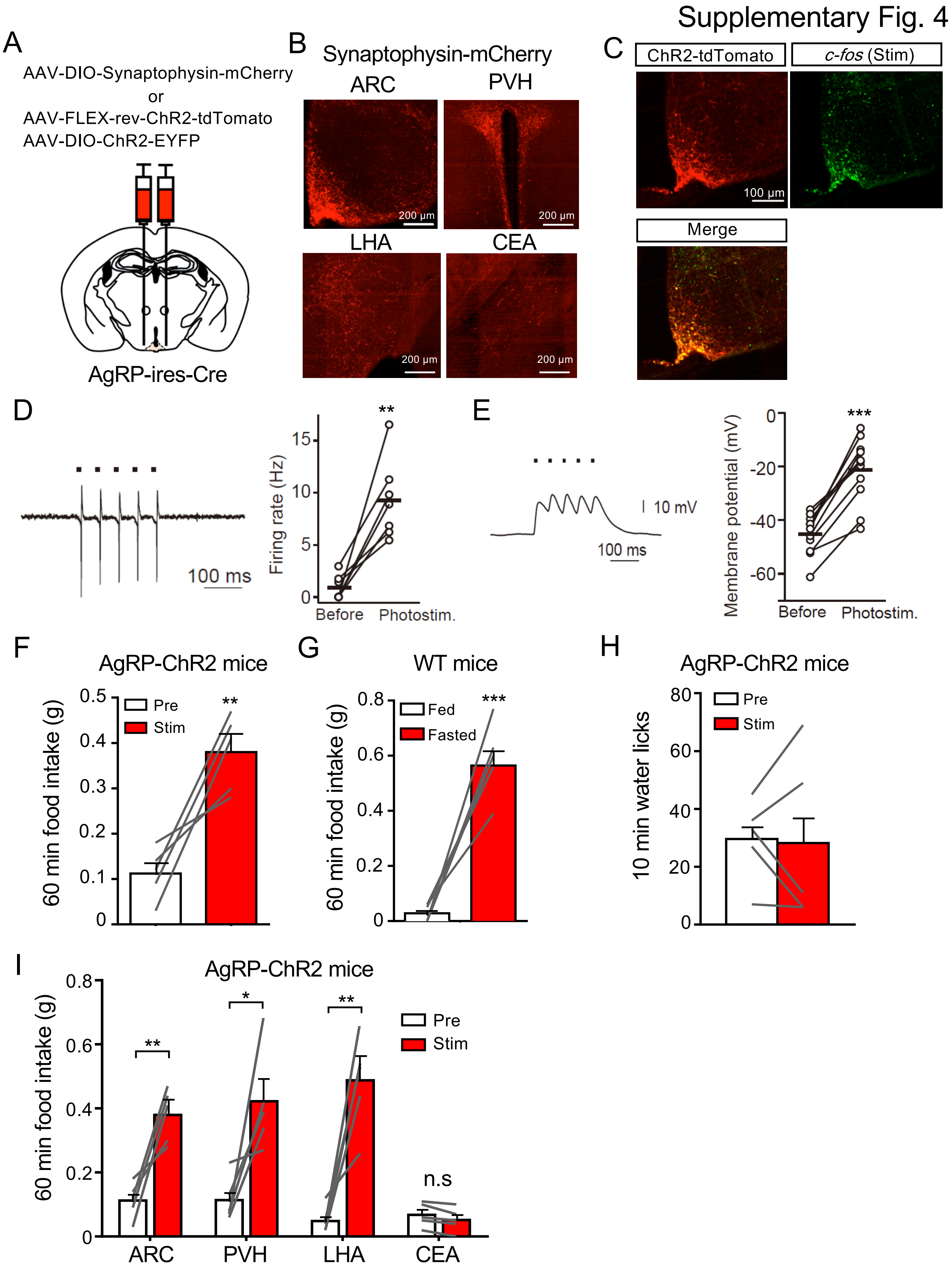
Supplementary Fig. 4
A
AAV-DIO-Synaptophysin-mCherry
or
AAV-FLEX-rev-ChR2-tdTomato
AAV-DIO-ChR2-EYFP
AgRP-ires-Cre
B
Synaptophysin-mCherry
ARC
PVH
LHA
CEA
200 μm
C
ChR2-tdTomato
c-fos (Stim)
100 μm
Merge
D
100 ms
Firing rate (Hz)
Before
Photostim.
E
10 mV
100 ms
Membrane potential (mV)
Before
Photostim.
F
AgRP-ChR2 mice
60 min food intake (g)
Pre
Stim
G
WT mice
60 min food intake (g)
Fed
Fasted
H
AgRP-ChR2 mice
10 min water licks
Pre
Stim
I
AgRP-ChR2 mice
60 min food intake (g)
Pre
Stim
n.s
ARC
PVH
LHA
CEA

Supplementary Fig. 4 Optogenetic investigation revealed orexigenic and non-orexigenic axon terminals of AgRP neurons.

(A) Injection of AAV-DIO-synaptophysin-mCherry, AAV-FLEX-rev-ChR2-tdTomato, AAV-DIO-ChR2-EYFP into the ARC of AgRP-ires-Cre mice. (B) Representative image of synaptophysin-mCherry expression in the ARC, PVH, LHA, and CEA in AgRP-ires-Cre mice injected with the AAV encoding Cre-dependent synaptophysin-mCherry. (C) Representative image of ChR2-tdTomato expression in the ARC of AgRP-ires-Cre mice injected with the AAV encoding Cre-dependent ChR2-tdTomato. *c-fos* expression was observed after 1 h laser stimulation. (D) A representative example of action potentials in ChR2-YFP expressing AgRP neurons in an acute slice. The duration of 5 blue-light stimulation at 20 Hz is indicated by the bars (left). A comparison of the spontaneous and photostimulation-evoked firing rates in individual neurons. The bar indicates the mean value for 7 neurons (right).

(E) A representative example of the membrane potential during the photostimulation (left). The membrane potentials before and during photostimulation in individual neurons. The bar indicates the mean value for 11 neurons (right).

(F) 1 h food intake during photostimulation of the ARC in AgRP-ChR2 mice.

(G) 1 h re-feeding response after overnight fasting in WT mice.

(H) Number of water licks (within 10 min) during photostimulation in AgRP-ChR2 mice. (I) Food intake was measured for 1 h in the presence or absence of photostimulation of the ARC (soma), PVH-projecting, LHA-projecting, or CEA-projecting AgRP neurons in AgRP-ChR2 mice. Increased food intake was observed when the ARC, PVH-projecting or LHA-projecting AgRP neurons were selectively activated. By contrast, such an increase was not observed when activating CEA-projecting AgRP neurons. All experiments were carried out with 8-16 week-old male mice. Data are given as means ± SEM. *$P<0.05$, **$P<0.01$, ***$P<0.001$. Statistical analysis was performed using a paired Student's *t*-test.

Supplementary Fig. 5

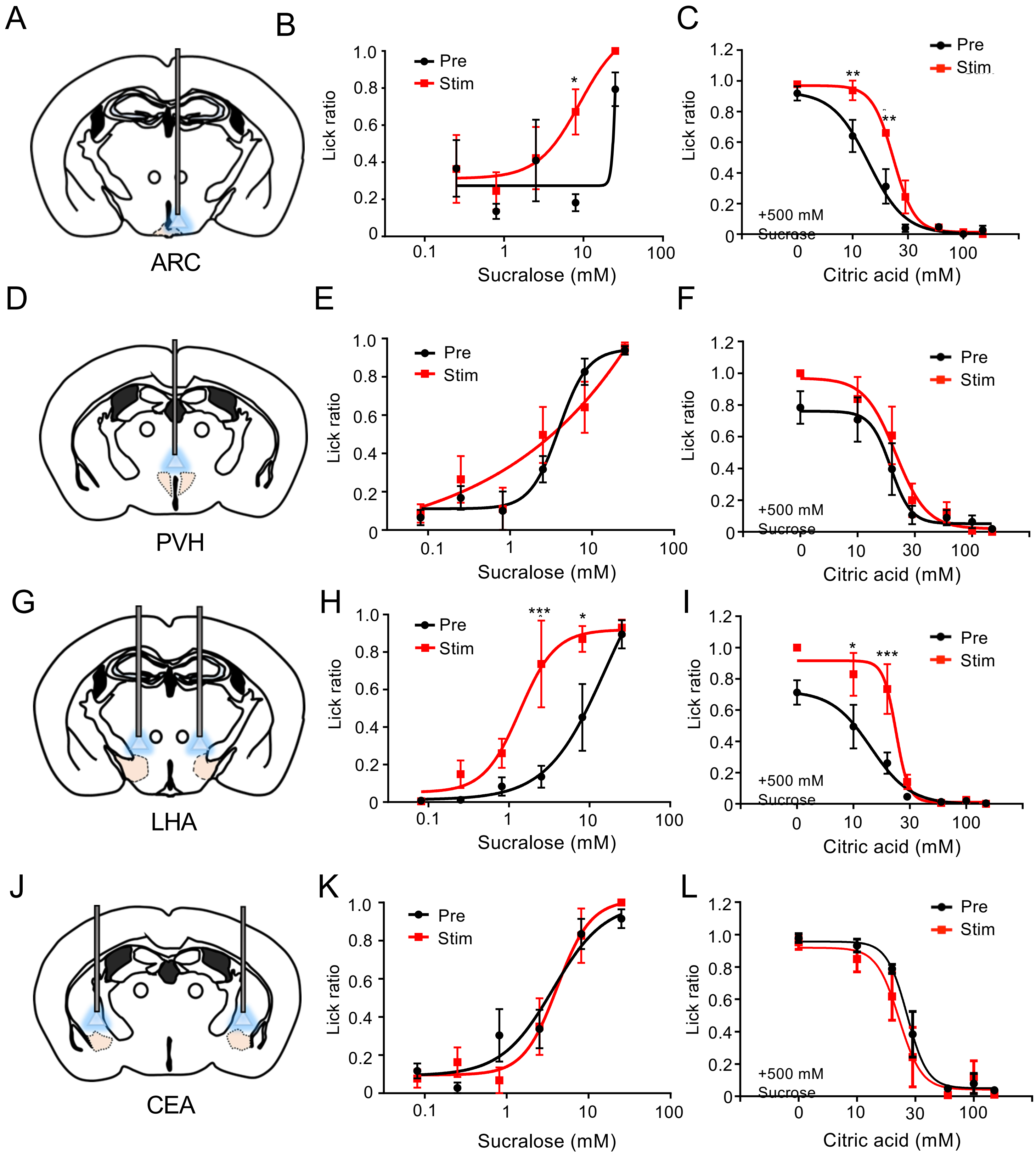

Supplementary Fig. 5 Brief access taste test for sucralose and citric acid solution when optogenetically activating the ARC (soma) (A–C), PVH-projecting (D–F), LHA-projecting (G–I), and CEA-projecting AgRP (J–L) neurons in AgRP-ChR2 mice. n=6, F=4.113, P=0.0496 in (B), n=6, F=15.59, P=0.0002 in (C), n=6, F=0.165, P=0.686 in (E), n=6, F=2.836, P=0.098 in (F), n=5, F=16.27, P=0.0002 in (H), n=5, F=16.96, P=0.0001 in (I), n=5, F=0.03, P=0.8637 in (K), n=5, F=1.899, P=0.174 in (L), two-way ANOVA with Bonferroni *post hoc* test. All experiments were carried out with 10- to 16-week-old male mice. Data are given as means ± SEM. *P<0.05, **P<0.01.

Supplementary Fig. 6

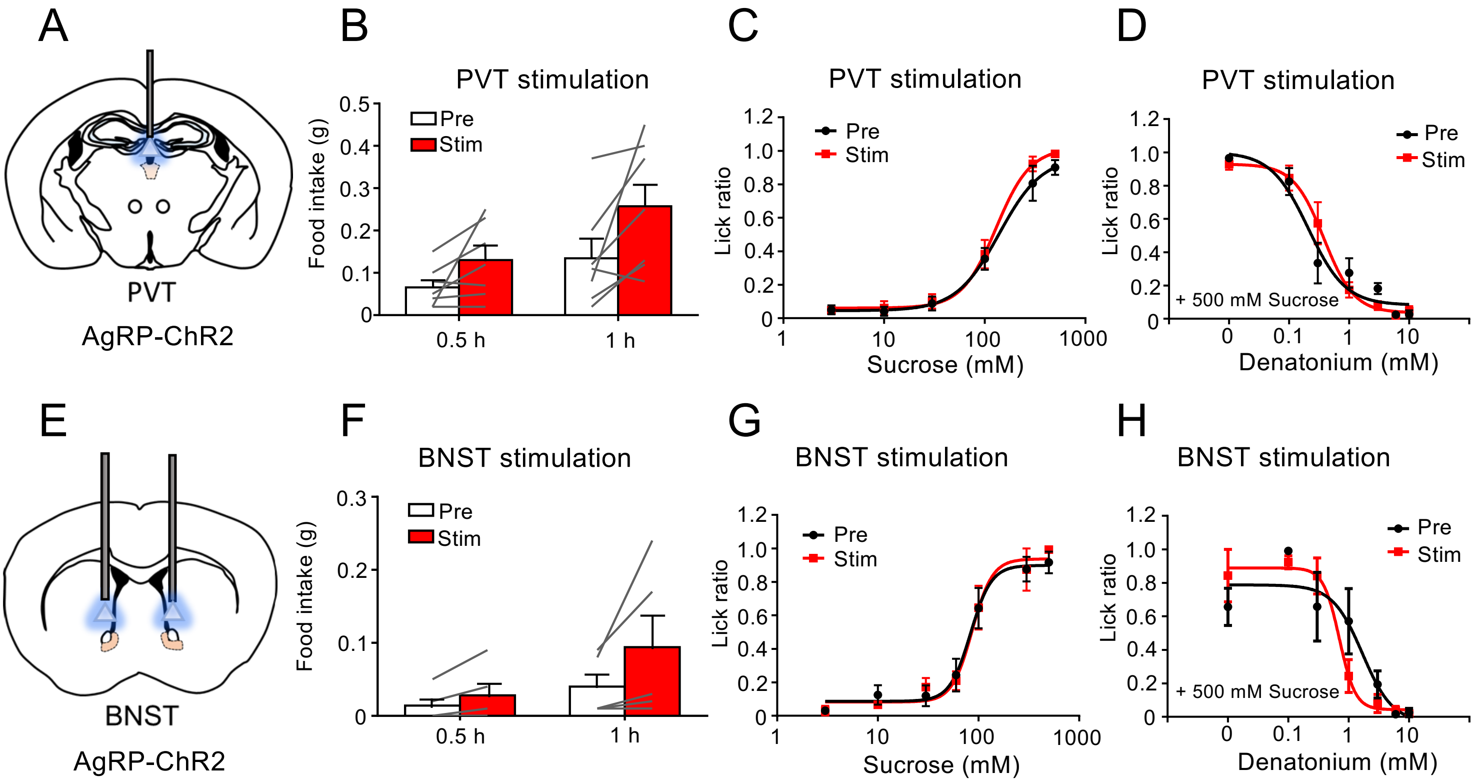

Supplementary Fig. 6 Selective activation of PVT-projecting or BNST-projecting AgRP neurons does not lead to a change in taste preference in AgRP-ChR2 mice.
(A) Representative image of the optic fiber implantation in the PVT. (B–D) 1h food intake (B), sucrose taste preference (C), and bitter taste preference (D) when optogenetically activating PVT-projecting AgRP neurons in AgRP-ChR2 mice. n=6, paired Student's *t*-test in (B). n=6, *F*=1.716, *P*=0.196 in (C) and n=6, *F*=0.019, *P*=0.891 in (D) , two-way ANOVA with Bonferroni *post hoc* test. (E) Representative image of the optic fiber implantation in the BNST. (F–H) 1 h food intake (F), sucrose taste preference (G), and bitter taste preference (H) when optogenetically activating BNST-projecting AgRP neurons in AgRP-ChR2 mice. n=6, paired Student's *t*-test in (F). n=7, *F*=0.016, *P*=0.898 in (G) and n=7, *F*=1.72, *P*=0.75 in (H), two-way ANOVA with Bonferroni *post hoc* test. All experiments were carried out with 8- to 16-week-old male mice. Data are given as means ± SEM.

Supplementary Fig. 7

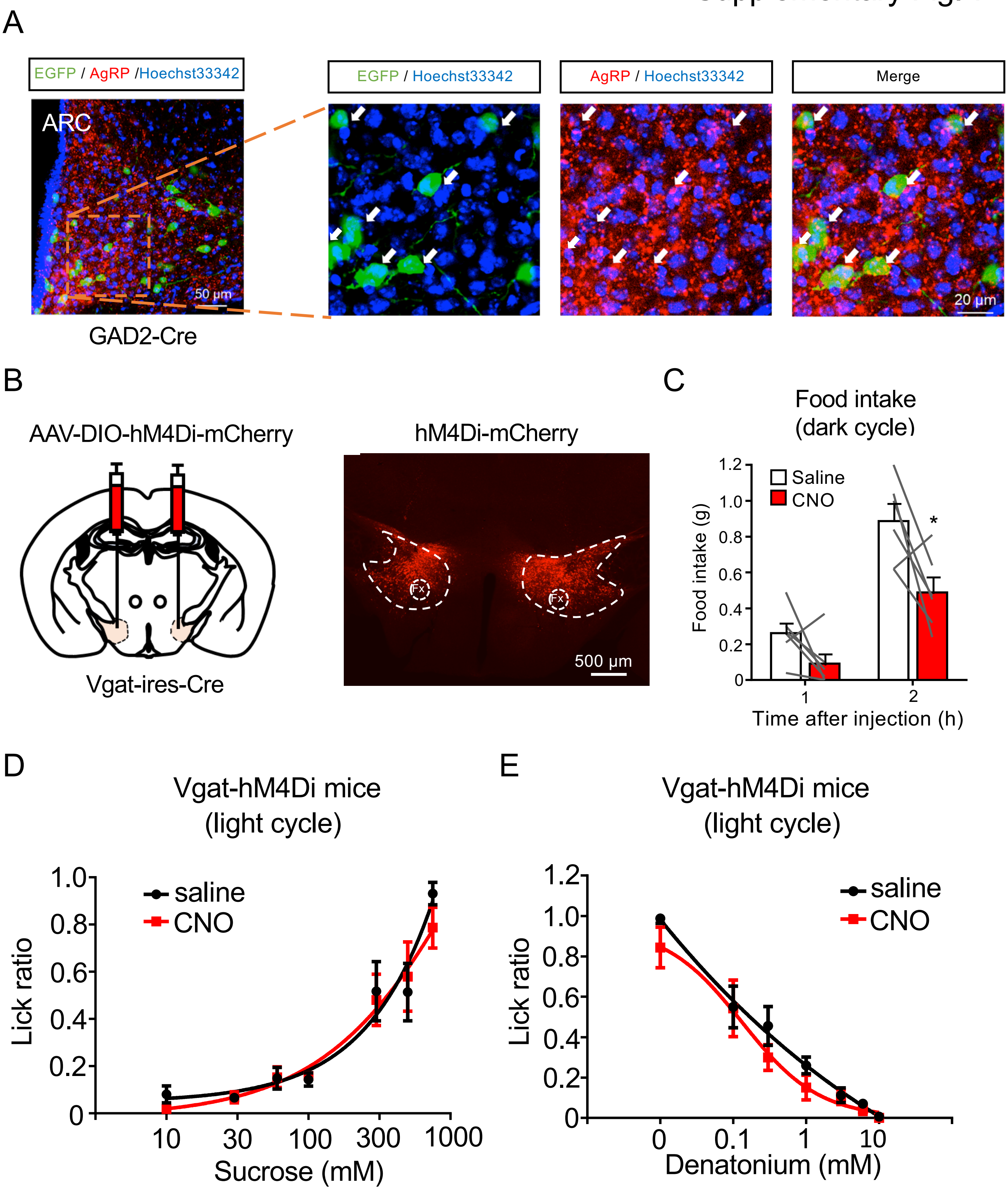

Supplementary Fig. 7 GABAergic neurons in the LHA connect to AgRP neurons but are not necessary for hunger-induced taste modification.

(A) Monosynaptic rabies tracing of GABAergic neurons in the LHA. Enlarged view shows parts of AgRP neurons (red) monosynaptically connected to GABAergic LHA neurons. (B) Bilateral injection of AAV encoding Cre-dependent hM4Di-mCherry into the LHA of Vgat-ires-Cre mouse (left) and a representative image of expression of hM4Di-mCherry in LHA (right). Fx, fornix. (C) Chemogenetic inhibition (CNO 1.0 mg/kg i.p.) of Vgat neurons in the LHA suppressed food intake in Vgat-hM4Di mice within 2 h from the beginning of the dark cycle. n=6, paired Student's *t*-test. (D and E) Brief access taste tests with sweet (D) and bitter (E) taste solutions in Vgat-hM4Di mice in the presence or absence of CNO (1.0 mg/kg i.p). n=6, F=0.311, P=0.579 in (D) and n=6, F=3.331, P=0.072 in (E), two-way ANOVA with Bonferroni *post hoc* test. Data are given as means ± SEM. *P<0.05.

Supplementary Fig. 8

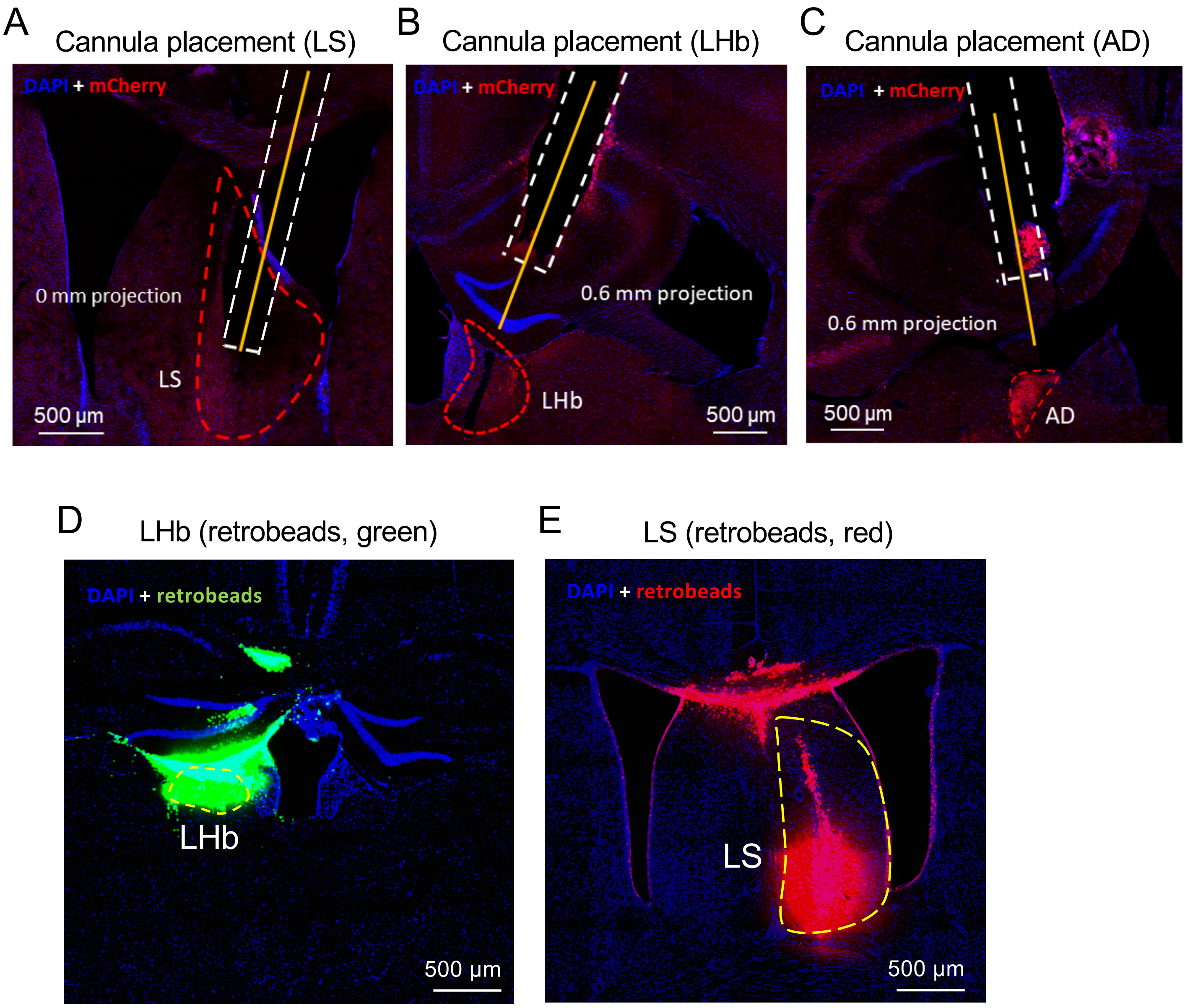

Supplementary Fig. 8 Cannula placement in LS, LHb and AD and Retrobeads infusion in LHb and LS.

(A-C) Representative Vglut2-hM4Di mice brain slice image showing the location of the guide cannula (white dashed line) and the assumed internal cannula in the LS, LHb and AD. The 0.6 mm projected internal cannula is used for LHb and AD and the non-projected internal cannula is used for LS.

(D-E) Representative image showing infused retrobeads (green) in the LHb (D) and retrobeads (red) in the LS (E).

Supplementary Fig. 9

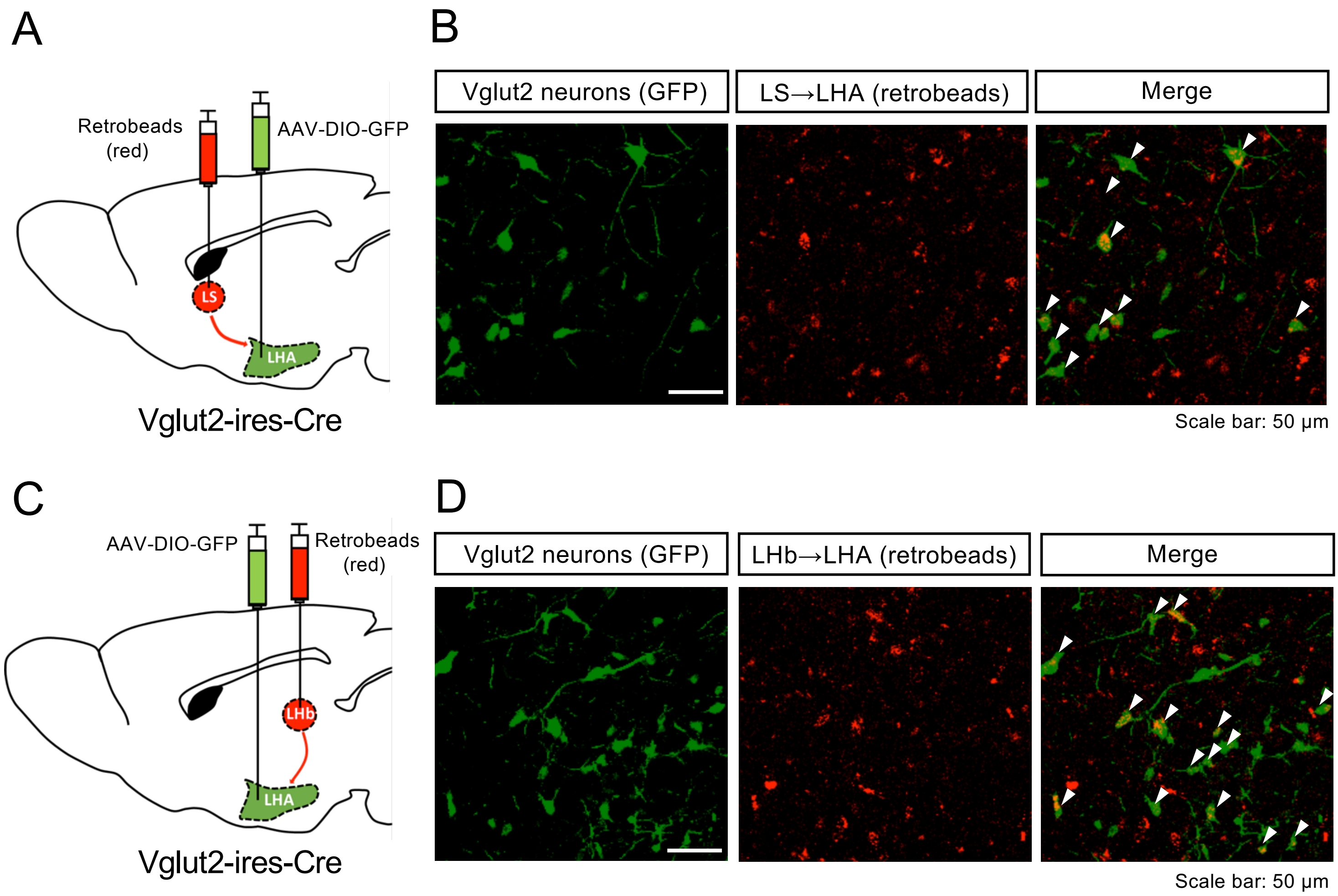

Supplementary Fig. 9 Some of the LS-projecting or LHb-projecting LHA neurons are glutamatergic.
(A) Schematic image of unilateral injection of AAV-DIO-GFP into the LHA and injection of retrobeads into the LS in Vglut2-ires-Cre mouse. (B) Representative confocal image showing GFP-labelled Vglut2 neurons and LS-projecting neurons in the LHA. Arrowheads indicate neurons co-labeled with both GFP and red retrobeads in the LHA. (C) Schematic image of unilateral injection of AAV-DIO-GFP into the LHA and injection of retrobeads into the LHb of Vglut2-ires-Cre mice. (D) Representative confocal image showing GFP-labeled Vglut2 neurons and LHb-projecting neurons in the LHA. Arrowheads indicate neurons co-labeled with both GFP and red retrobeads in the LHA.
