# Supplementary material for: Hypothalamic neuronal circuits regulating hunger-induced taste modification
Source: Nat Commun. 2019 Oct 8;10:4560. doi: 10.1038/s41467-019-12478-x (PMC6783447; doi:10.1038/s41467-019-12478-x)
Supplement: Supplementary file 3 — Reporting Summary [file 41467_2019_12478_MOESM3_ESM.pdf]

nature research

Corresponding author(s): Ken-ichiro Nakajima

Last updated by author(s): Sep 13, 2019

Reporting Summary

Nature Research wishes to improve the reproducibility of the work that we publish. This form provides structure for consistency and transparency in reporting. For further information on Nature Research policies, see Authors & Referees and the Editorial Policy Checklist.

Statistics

For all statistical analyses, confirm that the following items are present in the figure legend, table legend, main text, or Methods section.

n/a	Confirmed	
☐	☒	The exact sample size (*n*) for each experimental group/condition, given as a discrete number and unit of measurement
☐	☒	A statement on whether measurements were taken from distinct samples or whether the same sample was measured repeatedly
☐	☒	The statistical test(s) used AND whether they are one- or two-sided<br>*Only common tests should be described solely by name; describe more complex techniques in the Methods section.*
☒	☐	A description of all covariates tested
☐	☒	A description of any assumptions or corrections, such as tests of normality and adjustment for multiple comparisons
☐	☒	A full description of the statistical parameters including central tendency (e.g. means) or other basic estimates (e.g. regression coefficient) AND variation (e.g. standard deviation) or associated estimates of uncertainty (e.g. confidence intervals)
☐	☒	For null hypothesis testing, the test statistic (e.g. *F*, *t*, *r*) with confidence intervals, effect sizes, degrees of freedom and *P* value noted<br>*Give P values as exact values whenever suitable.*
☒	☐	For Bayesian analysis, information on the choice of priors and Markov chain Monte Carlo settings
☒	☐	For hierarchical and complex designs, identification of the appropriate level for tests and full reporting of outcomes
☒	☐	Estimates of effect sizes (e.g. Cohen's *d*, Pearson's *r*), indicating how they were calculated

Our web collection on statistics for biologists contains articles on many of the points above.

Software and code

Policy information about availability of computer code

Data collection	*Provide a description of all commercial, open source and custom code used to collect the data in this study, specifying the version used OR state that no software was used.*
Data analysis	GraphPad Prism 6

For manuscripts utilizing custom algorithms or software that are central to the research but not yet described in published literature, software must be made available to editors/reviewers. We strongly encourage code deposition in a community repository (e.g. GitHub). See the Nature Research guidelines for submitting code & software for further information.

Data

Policy information about availability of data

All manuscripts must include a data availability statement. This statement should provide the following information, where applicable:

- Accession codes, unique identifiers, or web links for publicly available datasets
- A list of figures that have associated raw data
- A description of any restrictions on data availability

The dataset of the current study are available from the corresponding author on reasonable request.

Field-specific reporting

Please select the one below that is the best fit for your research. If you are not sure, read the appropriate sections before making your selection.

☒ Life sciences ☐ Behavioural & social sciences ☐ Ecological, evolutionary & environmental sciences

Life sciences study design

All studies must disclose on these points even when the disclosure is negative.

Sample size	Sample sizes were based on previous studies that use similar methods. The number of animals for experiments were kept minimum according to local animal use policies.
Data exclusions	We excluded the data from the mice that do not show appropriate viral expression. This criteria was pre-established.
Replication	All experiments were repeated with similar results as indicated in figure legends.
Randomization	For behavioral experiments, we used male mice with similar age. For staining experiments, we used both male and female mice.
Blinding	For practical reasons, the investigators were not blinded to allocation during experiments and data analysis.

Reporting for specific materials, systems and methods

We require information from authors about some types of materials, experimental systems and methods used in many studies. Here, indicate whether each material, system or method listed is relevant to your study. If you are not sure if a list item applies to your research, read the appropriate section before selecting a response.

Materials & experimental systems

n/a	Involved in the study	
☐	☒	Antibodies
☒	☐	Eukaryotic cell lines
☒	☐	Palaeontology
☐	☒	Animals and other organisms
☒	☐	Human research participants
☒	☐	Clinical data

Methods

n/a	Involved in the study	
☒	☐	ChIP-seq
☒	☐	Flow cytometry
☒	☐	MRI-based neuroimaging

Antibodies

Antibodies used	Goat anti-AgRP (GT15023, 1:1000, Neuromics), rabbit anti-AgRP (G-003-57, 1:1000, Pheonix pharmaceuticals), goat anti-c-Fos (sc-52g, 1:400, Santa Cruz), and rat anti-RFP (5F8, 1:1000, ChromoTek) antibodies were used as primary antibodies. Donkey anti-goat Alexa fluor 488 (A-11055, 1:300, Life tech), donkey anti-rabbit Alexa fluor 488 (A-21206, 1:300, Life Tech), and donkey anti-Rat Alexa fluor 568 (ab175475, 1:300, abcam) antibodies were used as fluorophore-conjugated secondary antibodies.
Validation	The antibodies used in this study were validated by the manufacturers and did not show non-specific staining.

Animals and other organisms

Policy information about studies involving animals; ARRIVE guidelines recommended for reporting animal research

Laboratory animals	C57BL/6J (CLEA Japan), AgRP-ires-Cre (JAX-012899), Vglut2-ires-Cre (JAX-016963), Vgat-ires-Cre (JAX-016962). GAD2-ires-Cre (JAX-010802) mice were used. For behavioral experiments, only male animals were used. For immunohistochemistry, both male and female mice were used. Their ages were stated in the figure legends.
Wild animals	No wild animals were used in this study.
Field-collected samples	The study did not used samples collected from the field.
Ethics oversight	The animal experimental procedures followed the animal care guidelines approved by the University of Tokyo and by the National Institute for Physiological Sciences.

Note that full information on the approval of the study protocol must also be provided in the manuscript.
